# Photocatalytic fluoroalkylation by ligand-to-metal charge transfer

**DOI:** 10.3389/fchem.2024.1481342

**Published:** 2024-09-06

**Authors:** Jingyi Liu, Zhenwei Cui, Jingjing Bi, Xing He, Qingjie Ding, Hong Zhu, Chunhua Ma

**Affiliations:** ^1^ Collaborative Innovation Centre of Henan Province for Green Manufacturing of Fine Chemicals, Key Laboratory of Green Chemical Media and Reactions, Ministry of Education, Henan Engineering Research Centre of Chiral Hydroxyl Pharmaceutical, Henan Engineering Laboratory of Chemical Pharmaceutical and Biomedical Materials, School of Chemistry and Chemical Engineering, Henan Normal University, Xinxiang, China; ^2^ Chongqing Aoshe Bio-Chemical Co., Ltd., Chongqing, China; ^3^ School of Pharmacy, Xinyang Agricultural and Forestry University, Xinyang, Henan, China; ^4^ Anesthesiology and Perioperative, Xinxiang Central Hospital, Xinxiang, China

**Keywords:** trifluoromethylation, fluoroalkylation, visible-light-induced, LMCT, iron

## Abstract

Trifluoromethyl (CF_3_) and other fluoroalkyl groups are of great significance in the fields of pharmaceutical chemistry and agricultural chemicals. Fluoroalkyl acids, especially trifluoroacetic acid (TFA) is considered the most ideal fluoroalkylation reagent due to its low cost and easy availability. However, the extremely high oxidation potential requirement of TFA limits its wide application. In recent years, since visible-light-induced fluoroalkylation through the ligand-to-metal charge transfer (LMCT) process can overcome the above limitations, it has become an effective synthetic tool for the construction of fluorinated compounds with complex molecules and structures. In this review, according to the classification of different metal catalysts, we summarize the trifluoromethylation and fluoroalkylation of olefins, heteroaromatics, and terminal alkynes in different metal catalytic systems and their corresponding reaction mechanisms. The photocatalytic fluoroalkylation via LMCT is believed to expedite the development of fluoro-containing drugs, and more novel fluoroalkylation methologies using this strategy will be disclosed.

## 1 Introduction

**FIGURE 1:**
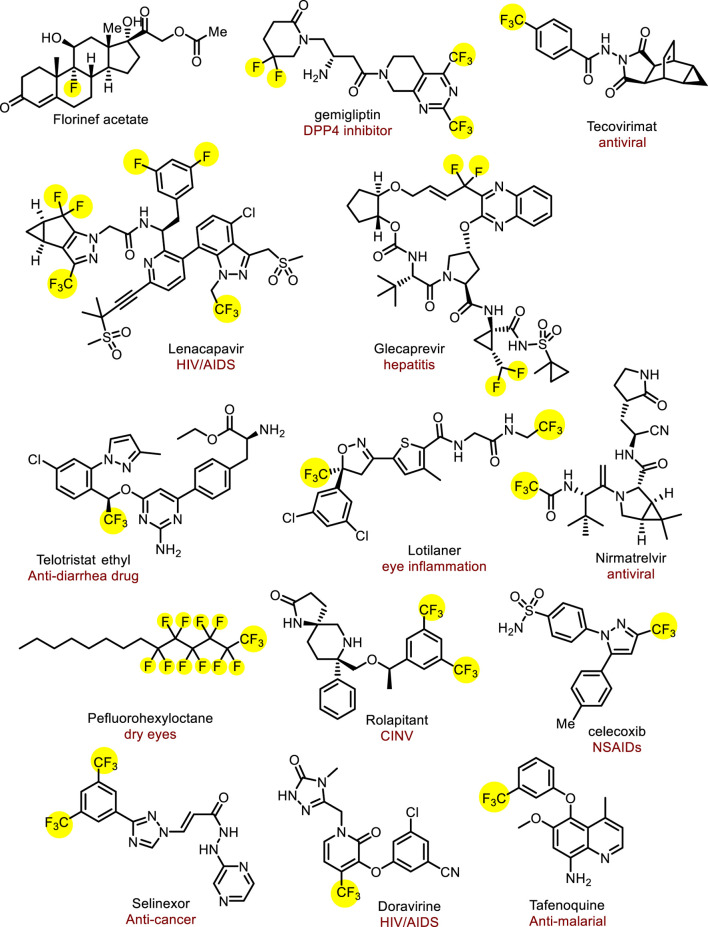
The pharmaceuticals containing fluoroalkyl groups.

The introduction of fluorine atoms into pharmaceuticals can improve their lipophilicity and membrane permeability, further affecting their metabolic stability and bioavailability (Gillis et al., 2015; Inoue et al., 2020; Han et al., 2021; Zhou et al., 2016; Purser et al., 2008; Chhetri et al., 2024). The first fluorine-containing drug was fludrocortisone, which was launched in 1954. Encouraged by the success of these drugs, the number of approved fluorine-containing drugs has stably increased over half a century (Inoue et al., 2020). A recent estimate suggests that over 20% of all marketed drugs have been fluorine-containing drugs and fluorinated compounds also account for about 30%–40% of agricultural chemicals; obviously, the proportion of fluoride drugs is increasing day by day (Figure 1) (Inoue et al., 2020; Chhetri et al., 2024; Shaw et al., 2024). Among them, fluoroalkyl, especially trifluoromethyl, is the most commonly used functional group in drug research and development, and widely exists in drugs. For example, a long-acting HIV-1 therapeutic and preventive drug lenacapavir approved in 2022, contains multiple trifluoromethyl and trifluoromethyl groups, which could endow the drug with low hepatic clearance (Marshall et al., 2024). Perfluorohexane, a 2023 newly approved drug to treat dry eye consists of perfluoroalkyl group and alkyl group (Ma et al., 2024). Therefore, the development of efficient fluoroalkylation methods has become a vital topic in medicinal chemistry and synthetic methodology.

**FIGURE 2:**
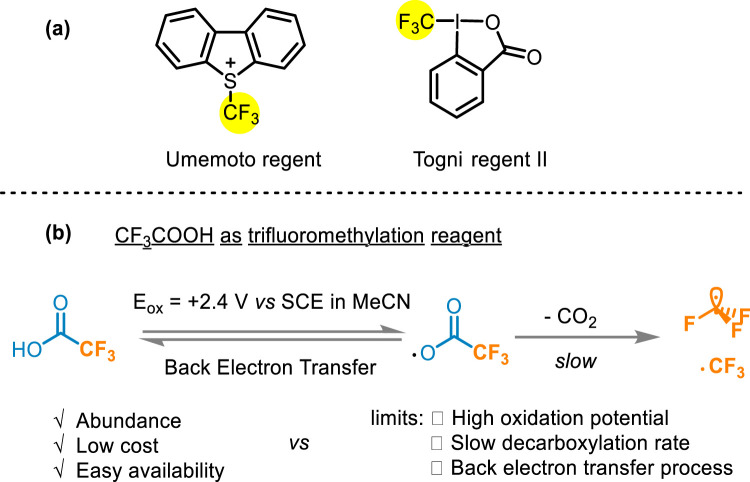
**(A)** Umemoto and Togni reagents; **(B)** Difficulties in using CF_3_COOH as a trifluoromethylation reagent.

Many fluoroalkylation reactions have been reported (Lalloo et al., 2021; Kuehl and Taylor, 2023; Jin et al., 2024; Pan et al., 2016; Tian et al., 2024; Liang et al., 2023). The early used reagents, such as CF_3_I (Kino et al., 2010), CF_3_SO_2_Cl (Kamigata et al., 1991), and CF_3_SO_2_Na (Langlois et al., 1991), have been constrained in their widespread applicability owing to excessively harsh conditions and instability (Kamigata et al., 1991; Langlois et al., 1991; Haszeldine, 1949; Bian et al., 2023). Currently developed commercially available electrophilic fluoroalkyl reagents such as Umemoto and Togni reagents (Figure 2A) have fallen short of ideal standards due to high oxidation characteristics, or other limitations liking high temperature and suboptimal atomic economy (Li et al., 2017; Zhao et al., 2010; Egami et al., 2015; Cheng and Yu, 2016; Umemoto and Ishihara, 1998; Umemoto and Ishihara, 1999; Jia et al., 2021). Compared with these two common CF_3_ sources, TFA is generally considered an ideal source of trifluoromethyl due to its abundance, low cost, and easy availability (Lopez and Salazar, 2013). However, the oxidation potential requirement of TFA is relatively high [>2.2 V vs. saturated calomel electrode (SCE)] (Figure 2B), leading to the need for forced conditions to release the CF_3_ group (Depecker et al., 1999). Traditionally metal-catalyzed fluoroalkylation methods rely on oxidant and guiding groups to activate the C−H bond, what’s more, the high electronegativity of R_F_ groups would cause slow reduction elimination at high temperatures (Shaw et al., 2024). In addition, previous hydrogen difluoromethylation methods also show range tolerance and complex multi-step synthesis (Bian et al., 2023; Lin et al., 2016; Tang et al., 2015). Hence, the development of methods using trifluoroacetic acid or other fluoroalkyl acid as fluoroalkyl radical sources to achieve the fluoroalkylation modification is an urgent problem in organic chemistry.

**FIGURE 3:**
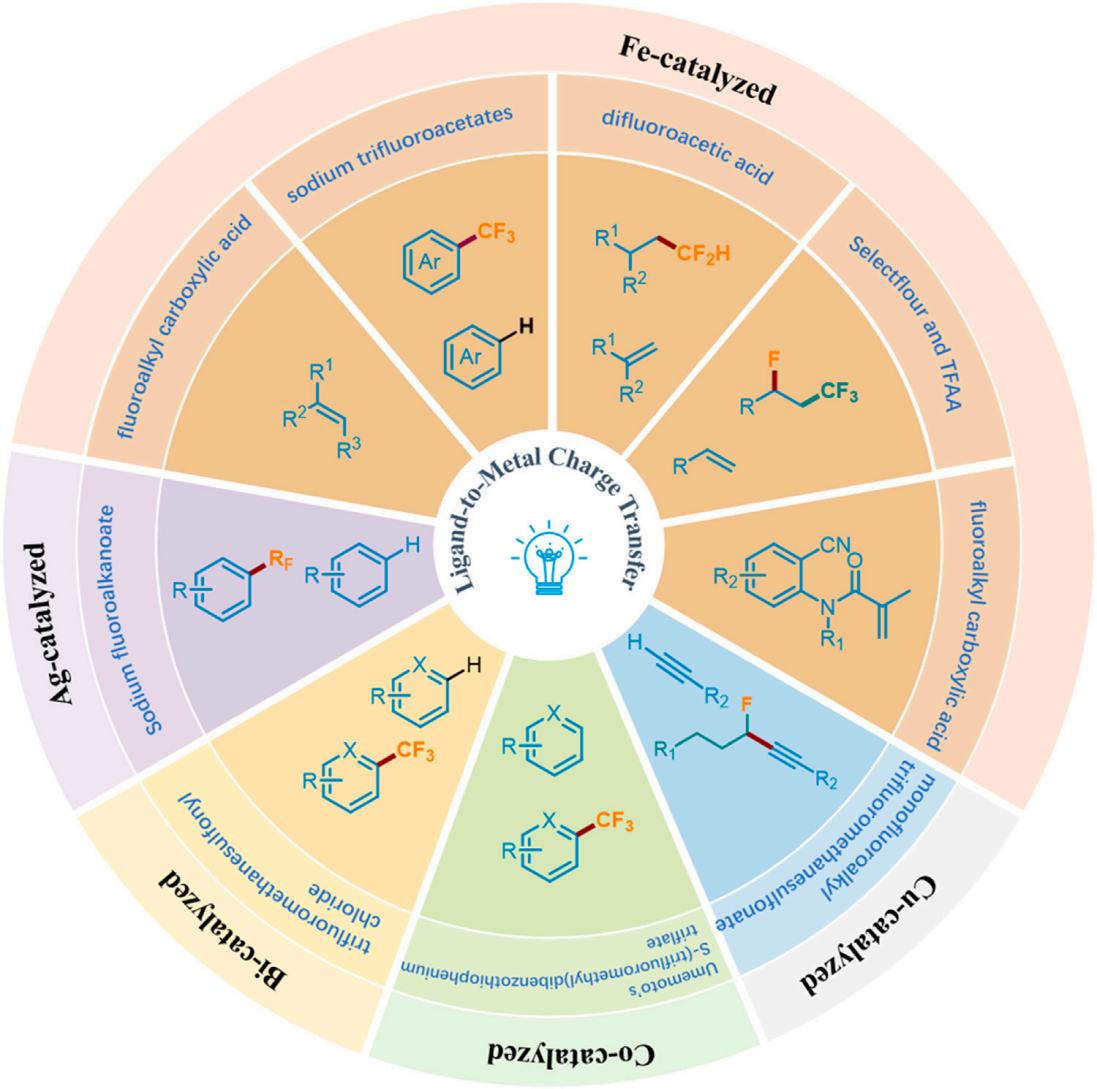
Photocatalytic fluoroalkylation by ligand-to-metal charge transfer.

Recently, photocatalysis has become a paradigm shift for organic chemistry, achieving diverse chemical transformations (Ma et al., 2022a; Ma et al., 2022b; Ma et al., 2022c; Ma et al., 2022d; Ma et al., 2021; Ma et al., 2023; Li et al., 2024; Dinda et al., 2024; Biswas et al., 2023; Wu and Huang, 2023; Ji et al., 2024; Chiappini et al., 2024; Zhang and Gevorgyan, 2024; Wen et al., 2023; Chandrappa et al., 2023). Wherein, the visible-light-induced LMCT process has proved to be a powerful and efficient organic synthesis tool for the construction of valuable molecules by obtaining the required active electrophilic radicals (including carboxylate) from metal complexes (Birnthaler et al., 2023; Abderrazak et al., 2021). Generally, this process is considered to have two steps: visible light radiation induces intramolecular electron transfer from ligand to metal center to promote the formation of the metal center, and then generates oxidative ligand radical dissociation (May and Dempsey, 2024). Traditionally, precious and rare elements have dominated inorganic photophysical and photochemical processes, but now they are transforming towards cheaper and richer transition metals (Fe, Cu, Ni, Co, etc.), which exhibit surprising reactivity and photoluminescence behavior in electron-excited states (Wegeberg and Wenger, 2021). The emergence of LMCT makes it possible to solve the problem of converting fluoroalkyl acids to fluoroalkyl radicals under mild conditions. Accordingly, combining the visible light-induced homolysis (VLIH) concept with transition metal-based photocatalysts can surpass the limitations of traditional photocatalysts (Abderrazak et al., 2021). Although Ranjay Shaw et al. reviewed the C−H bond trifluoromethylation and fluoroalkylation, they mainly made photocatalytic and electrochemical methods (Shaw et al., 2024). There are few reviews focusing on fluoroalkylation based on visible-light-induced LMCT. In this minireview, we comprehensively summarize the fluoroalkylation of olefins, aromatics or heteroaromatics, and terminal alkynes catalyzed by metal complexes under visible light irradiation, and classified them according to the types of metals involved (Figure 3).

## 2 Photoinduced fluoroalkylation by LMCT

### 2.1 Iron-catalyzed fluoroalkylation by LMCT

**SCHEME 1:**
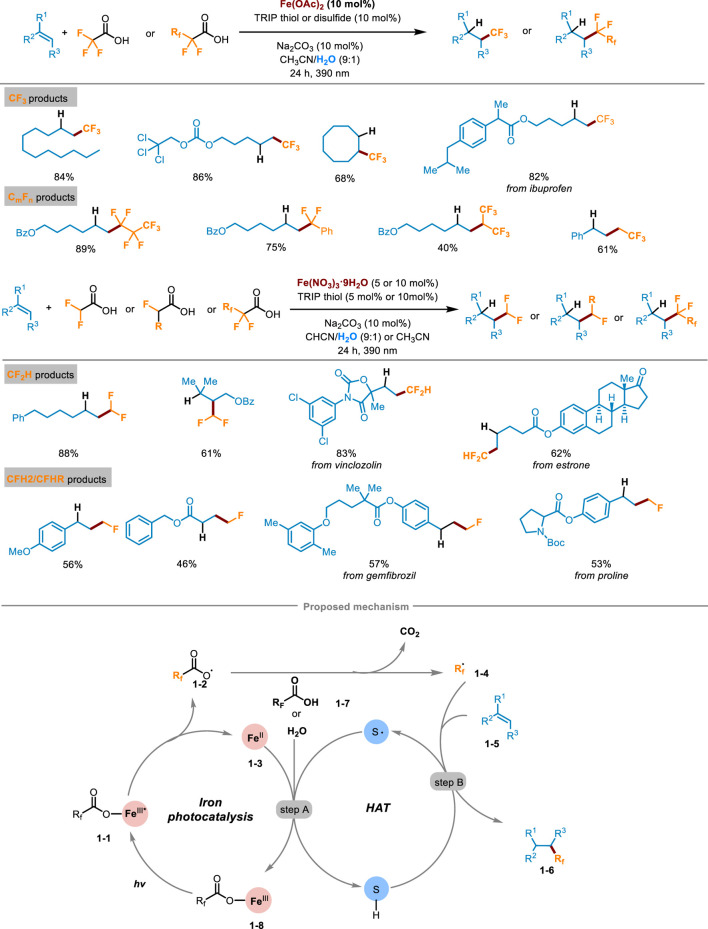
Photocatalytic hydrofluoroalkylation of alkenes with carboxylic acids.

The fluoroalkylation reactions of alkenes represent highly effective tools for delivering various fluoro-containing compounds. In 2023, West’s research group at *Rice University* reported a visible-light-induced fluoroalkylation of unactivated olefins using cheap and easily available fluoroalkyl carboxylic acid as the only fluoroalkyl radical source, which realized the hydrofluoroalkylation (including trifluoro-, difluoro-, monofluoro- and perfluoroalkylation) of alkenes through the dual-catalytic synergism of iron photocatalysis and redox active thiol catalyzed hydrogen-atom-transfer (HAT) process (Scheme 1) (Bian et al., 2023). The method demonstrated high compatibility with a wide array of alkenes with different functional groups, and the corresponding products were obtained in medium to good yields. A series of natural products or drug-derived alkenes could also be successfully compatible with this transformation, demonstrating the potential effectiveness of the late-stage modification. The deuterium labeling study by replacing H_2_O with D_2_O showed the deuterotrifluoromethylated product could be afforded in a high deuterium incorporation rate which confirmed that hydrogen was provided by the cosolvent H_2_O. A sequence of mechanism studies were undertaken to elucidate the underlying reaction pathway of this transformation. Initially, when the radical scavenger 2,2,6,6-tetramethyl-1-piperidinoxy (TEMPO) was introduced into the reaction system, the formation of products was completely inhibited and almost all the alkene starting materials were recovered. What’s more, the presence of TEMPO−CF_3_ adduct indicated radical intermediates involved in the reaction. Subsequently, the authors investigated the reaction employing two distinct substrates capable of eliciting a radical clock process. The experimental results also demonstrated the involvement of radical intermediates. Finally, the parallel kinetic isotope effect (KIE) experiment pinpointed the HAT process as the rate-determining step of this transformation. Guided by these investigations, the following mechanistic pathway was proposed in Scheme 1. Firstly, the fluoroalkyl carboxylic acid chelated with Fe(II) to generate Fe(III) complex **1**-**1**, which was excited under visible light irradiation. Then LMCT occurred in the excited iron complex to cause homolytic cleavage of the O−Fe bond, affording carboxyl radicals **1-2** and low-valent Fe^II^ species **1-3**. After removing one molecule of CO_2_ from **1-2**, the key fluorinated alkyl radical **1-4** was generated. It then was captured by alkenes **1-5**, affording a transient alkyl radical intermediate. Then, a HAT process occurred between the thiol cocatalyst and alkyl radical intermediate, gaining the desired hydrofluoroalkylation product **1-6** and thiol radicals (step B). At last, the thiyl radical further oxidized Fe^II^ **1-3** to release high-valent Fe^III^ species **1-8**, simultaneously obtaining a proton from another carboxylic substrate **1-7** or H_2_O, closing both the iron photocatalysis cycle and redox active thiol catalytic cycle (step A).

**SCHEME 2:**
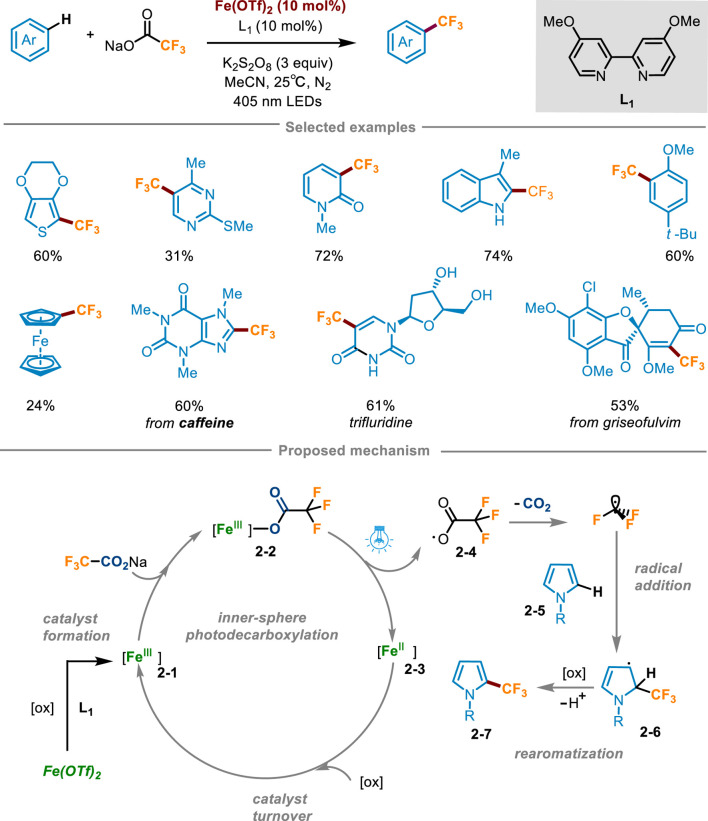
Trifluoromethylation of (hetero)arenes enabled by iron photocatalysis mediated decarboxylation of trifluoroacetate.

Trifluoroacetates are cheap and easily available trifluoromethyl sources, which play a crucial role in pharmaceuticals and agricultural chemicals. However, trifluoroacetate has a high oxidation potential (+2.4 V vs. SCE in MeCN) (Depecker et al., 1999), which makes it hard to generate trifluoromethyl radical through decarboxylation reaction to participate in the subsequent reaction. In 2023, Francisco’s group developed a direct C−H trifluoromethylation of various electron-rich (hetero)aromatics through iron(II) trifluomethanesulfonate Fe(OTf)_2_ catalyzed photodecarboxylation of trifluoroacetates (Scheme 2) (Fernández-García et al., 2024). The optimal reaction conditions were determined as follows: (hetero)arene reacted with sodium trifluoroacetate (4–6 equiv) in the presence of Fe(OTf)_2_ (10 mol%) and 4,4′-dimethoxy-2,2′-bipyridine (**L1**, 10 mol%) as the active catalyst, K_2_S_2_O_8_ (3 equiv.) as the oxidant, acetonitrile (0.1 M) as the solvent under the irradiation of 405 nm LED. Oxidants did not involve in the decarboxylation process, but were crucial for the conversion of Fe(II) to catalytically active Fe(III) species. Substrate scope investigation revealed that a range of pyrroles with *N*-aromatic or aliphatic substitution, thiophene, pyridine, pyrimidine, indoles, and arenes with an electron donating group could achieve direct functionalization via this reaction. It was worth noting that this scheme also allowed for direct trifluoromethylation of ferrocene, which was difficult to access by the previous methods. The utility of this protocol was highlighted by the successful last-stage trifluoromethylation of drugs, agrochemicals and natural products. For instance, caffeine, pentoxifylline, theophylline, uracil, protected pyrimidine nucleobases, antifungal griseofulvin, muscle relaxant metaxalone, nonsteroidal anti-inflammatory indomethacin, and natural product melatonin could be converted into the corresponding trifluoromethylated products. Moreover, one of the largest-selling drugs trifluridine (Lonsurf^®^) could be afforded using this method from unprotected 2-deoxyuridine in a good yield. The radical trapping experiment offered definitive evidence for the mechanism of the reaction being a free radical process, whereas the UV-visible spectroscopic analysis corroborated the occurrence of a trifluoroacetic acid-mediated photodecarboxylation reaction of **2-3**. The mechanism was depicted in Scheme 2: at first, *in situ* coordination of Fe(OTf)_2_ with **L1**, followed by oxidation with K_2_S_2_O_8_ to form catalytically active Fe(III) species **2-1**. Subsequently, sodium trifluoroacetate coordinated with the Fe(III) substance **2-1**, resulting in the formation of Fe(III) carboxylic acid complex **2-2**. Under visible light irradiation at 405 nm, LMCT occurred to trigger Fe−O homolytic cleavage, furnishing Fe(II) species **2-3** and trifluoroacetic acid radical **2-4**, which then underwent decarboxylation to gain CF_3_ radical. Then CF_3_ radical was added to the substrate **2-5**, producing the radical intermediate **2-6** which was further converted into the trifluoromethylation product **2-7** after oxidation and deprotonation. Fe(II) **2-3** was oxidized by K_2_S_2_O_8_ to regenerate Fe(III) **2-1** and achieve the catalyst turnover.

**SCHEME 3:**
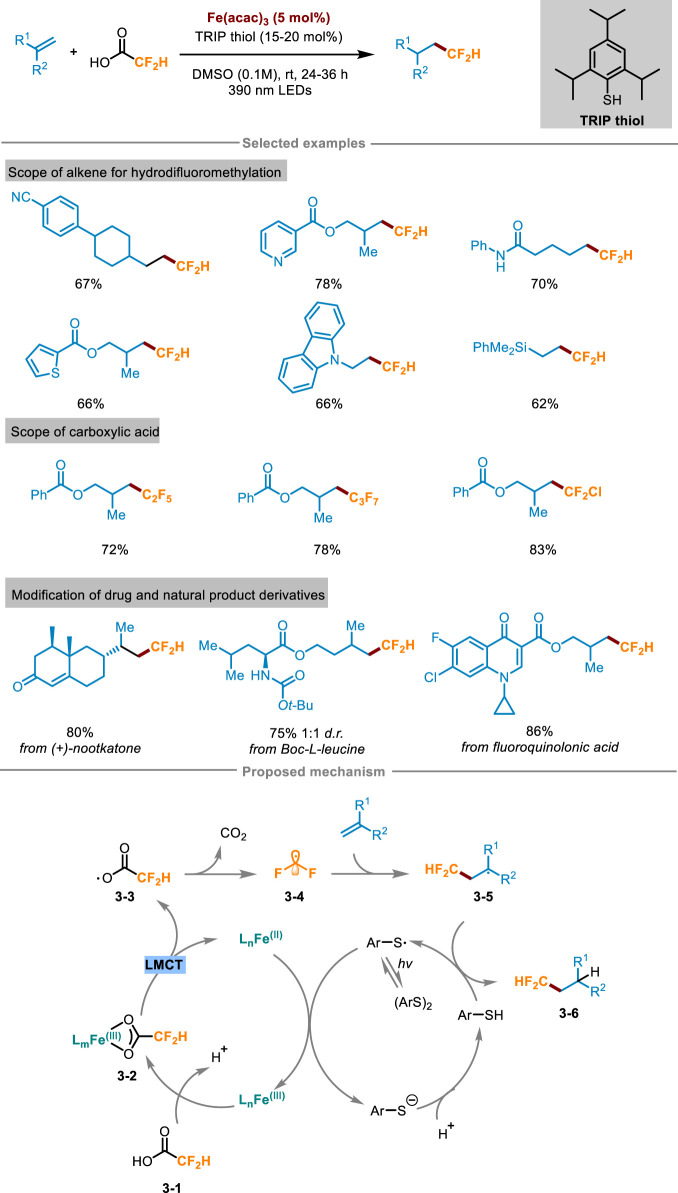
Photoinduced hydrodifluoromethylation of alkenes enabled by Fe-LMCT mediated decarboxylation.

Difluoroalkylation pathways of alkenes include halogen atom transfer (e.g., BrCF_2_H, ClCF_2_H) (Zhang et al., 2022; Bacauanu et al., 2018) single-electron oxidation or reduction (e.g., Zn(SO_2_CF_2_H)_2_ or ClSO_2_CF_2_H) (Fujiwara et al., 2012; Tang and Dolbier, 2015). However, most of the reported reactions also need to use expensive fluoroalkylation reagents, stoichiometric oxidants or additives, which are relatively not atomic economical. To overcome these limitations, Xia and Gou’s group presented a universal synthesis method in 2024 (Scheme 3) (Qi et al., 2024), which utilized visible light to induce ligand-to-iron charge transfer, and activated difluoroacetic acid to form difluoromethyl radical without adding additional stoichiometric oxidants, thus overcoming the high redox potential of fluoroalkyl carboxylic acid raw materials. The reaction system easily facilitated the difluoromethylation of unactivated alkenes, demonstrating its compatibility towards a diverse array of functional groups. Furthermore, it also exhibited excellent performance in the difluoromethylation of some pharmaceutical molecules, natural products, as well as bioactive molecular derivatives, showcasing its broad applicability and synthetic potential. For example, α-amino acid derivatives Boc-L-leucine can react with difluoroacetic acid with a yield of 75%. Likewise, the protocol has broad practicality, and (+)-nootkatone successfully reacted with difluoroacetic acid on a larger scale (4.0 mmol) with a yield of 80%. The reaction process was amplified using an expandable continuous flow reaction device, which greatly improved the reaction speed compared with the “single pot” method. A comprehensive suite of experiments, encompassing free radical trapping, free radical clock, isotope labeling, and electron paramagnetic resonance (EPR) spectroscopy, were conducted to gain a deeper understanding of the reaction mechanism, ultimately leading to the proposal of a plausible reaction pathway. As shown in Scheme 3, difluoroacetic acid **3-1** and Fe(acac)_3_ formed Fe(III)-carboxylate chromophore **3-2** *in situ*, and then the complex **3-2** was photoexcited to the LMCT state to obtain Fe(II) complex and carboxyl radical **3-3**, followed by decarboxylation to release the key intermediate difluoromethyl radical **3-4**. Subsequently, **3-4** engaged in radical addition with the substrate alkenes to gain radical intermediate **3-5**, then received a hydrogen atom from Ar-SH (Ar = 2,4,6-triisopropylphenyl) to yield the target product **3-6** and ArS radical intermediate. After this, ArS radical oxidized the Fe(II) complex to Fe(III) and regenerated Fe(III)-carboxylic acid complex **3-2** and ArS^−^, finally realizing the catalytic cycle.

**SCHEME 4:**
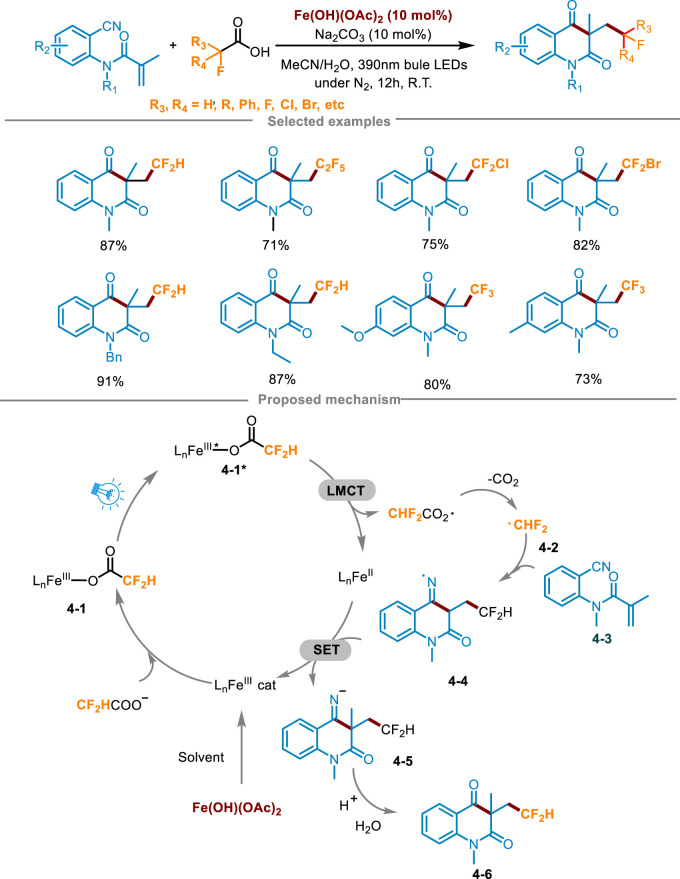
Photocatalytic synthesis via an iron-catalyzed LMCT decarboxylation process.

Currently, the synthesis of fluoroalkylated quinolinones still has strict requirements for reaction conditions, usually requiring expensive metal catalysts and excessive oxidants, and the choice of fluoroalkyl radical sources is also limited (Lin et al., 2017). To overcome these difficulties, Yang’s group developed a practical method for the efficient synthesis of fluoroalkylated quinolinones in 2024 (Scheme 4) (Song et al., 2024). The carboxylic acids underwent visible-light-driven LMCT process and decarboxylation to generate fluoroalkyl radicals, which reacted with *N*-(2-cyanophenyl)-*N*-methylacrylamides **4-3** via free radical addition/cyclization cascade process to access fluorinated nitrogen heterocyclic derivatives. The fluoroalkylated quinolinone **4-6** was synthesized in an 87% yield by combining **4-3**, difluoroacetic acid (5 equiv.), Fe(OH)(OAc)_2_ (0.02 mmol, 10 mol%) and Na_2_CO_3_ (10 mol%) in the mixture solvent of MeCN and H_2_O, under the condition of N_2_ atmosphere and irradiation with a 390 nm blue LED light for 12 h at ambient temperature. Optimization experiments indicated that when using other iron catalysts instead of Fe(OH)(OAc)_2_, it caused a decrease in yield. In the aspect of light source, increasing or decreasing the irradiation wavelength resulted in a decrease in the yield of the product. Control experiments proved that the photocatalyst, inert atmosphere, and light irradiation were essential for the transformation. Attempts at a gram-scale continuous flow reaction were also made using a photocatalytic device, and obtained the product with a moderate yield (66%), having a good application prospect. The study also showed the extensive reaction range of fluoroalkyl carboxylic acids. Most of these anionic acids with high oxidation potential can react smoothly and achieve excellent yields, including other acids with medium yields, which could prove that this method can effectively solve the problem of high oxidation potential. Simultaneously, it could also obtain the product of trifluoromethyl radical addition. The radical scavengers 2,6-di-tert-butyl-4-methylphenol (BHT) and TEMPO significantly inhibited the response, suggesting that the reaction may have undergone a free radical process. Furthermore, an isotope labeling experiment was conducted, involving the substitution of H_2_O with H_2_^18^O in the solvent, conclusively demonstrating that the carbonyl group originated from the hydrolysis of the imine. Drawing upon these findings and phenomena observed during the condition screening, a plausible reaction mechanism was elucidated in Scheme 4. Initially, Fe(OH)(OAc)_2_ formed a trivalent iron complex in the presence of the solvent CH_3_CN. Subsequently, it reacted with fluoroalkyl carboxylic acid anions to generate complex **4-1**, which was further converted into the excited state intermediate 4-2* under blue LED irradiation. Then, a key LMCT process occurred in this excited complex 4-2* through inner electron transfer, generating a Fe(II) complex (L_n_Fe^II^) and a carboxyl radical, which furnished the key fluoroalkyl radical **4-2** after decarboxylation. Then the fluoroalkyl radical combined with **4-3** to form imine radical intermediate **4-4**. An imine anion **4-5** was generated via a SET process between **4-4** and the Fe(II) complex, along with the regeneration of the trivalent iron complex to close the photoredox cycle. Finally, **4-5** rapidly abstracted a hydrogen proton to produce the imine hydrolyzed by water in the solvent to deliver the desired fluoroalkylated quinolinone **4-6**.

**SCHEME 5:**
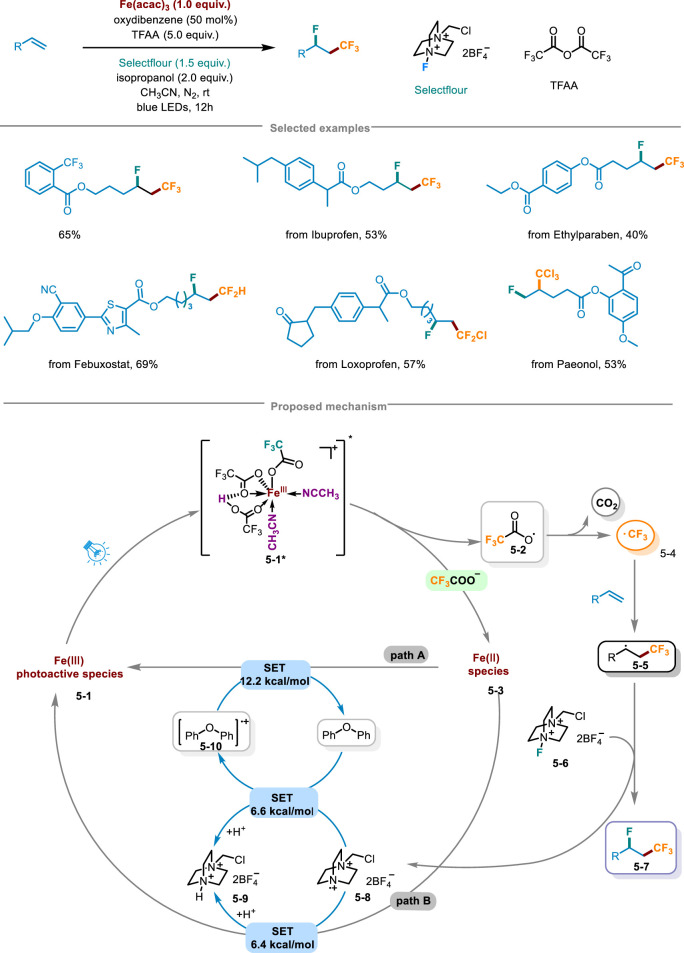
Fluoro-polyhaloalkylation of non-activated alkenes by Brønsted acid-unlocked iron LMCT.

In 2024, Niu and coworkers from *Zhengzhou University* proposed a innovative approach for fluoro-polyhaloalkylation of non-activated olefins unlocking the LMCT of iron optically active species by Brønsted acid, activating inert polyhalogenated carboxylic acid salts (C_n_X_m_COO^−^) to release polyhalogenated alkyl radicals, and achieved the fluoro-polyhaloalkylation of olefins through alkenes capture and radical fluorination (Scheme 5) (Jiang et al., 2024). By harnessing inexpensive trifluoroacetic anhydride (TFAA), isopropanol, oxygenated diphenyl and acetonitrile, a reaction system of Brønsted acid and trifluoroacetic acid was established. This system, when coupled with commercial Fe(acac)_3_ as the metal catalyst, Selecflour as the radical fluorinating agent, along with blue LED irradiation, enabled the successful execution of fluoro-trifluoromethylation reactions with exceptional regional selectivity and moderate to high yields. Further stoichiometric investigations revealed that under the irradiation of blue LED, Fe(CF_3_COO)_3_ unlocking and releasing CF_3_ radicals depended on the presence of a strong Brønsted acid, and only when Fe(CF_3_COO)_3_ was in acidic conditions, the photodegradation of Fe(CF_3_COO)_3_ occurred, highlighting the crucial role of the acid in facilitating this process. It was also found that the yield of fluoro-trifluoromethylation was determined by the amount of Brønsted acid, and the optimal condition for generating CF_3_ radicals was that it was three equivalents relative to the loading amount of Fe(CF_3_COO)_3_. The hydrogen bonding effect of Brønsted acid and the coordination of CH_3_CN solvent were crucial for ensuring the efficacious assembly of iron and C_n_X_m_COO radical light-harvesting species. Light on/off experiments confirmed the requirement of continuous irradiation in this pathway. The late-stage functionalization of pharmaceuticals has exhibited remarkable synthetic compatibilities, encompassing fluoro-polyhaloalkylation of ibuprofen, the germicide ethylparaben, as well as other intricate drug molecules. Furthermore, it had features like gram-scale synthesis and a low iron catalyst loading (TON = 160). Thereby the scheme presented potential application prospects in drug discovery and synthetic chemistry. The rational mechanism was as follows in Scheme 5: in the environment containing Brønsted acid, CF_3_COO^−^ and the solvent CH_3_CN, the light-harvesting species **5-1** would undergo a Brønsted acid-catalyzed unlocking of iron-mediated LMCT under light irradiation, further transferring CF_3_COO radical **5-2** and Fe(II) species **5-3**. Subsequently, radical intermediate **5-2** released CO_2_ to deliver CF_3_ radical **5-4**, which via radical addition obtained adduct **5-5**. Then **5-5** reacted with Selectfluor **5-6** by radical fluorination, leading to the generation of fluoro-trifluoromethylation product **5-7**. Under the regulation of the redox buffer oxygenated diphenyl, *N* radical cation **5-8** avoided the possible formation of related C-N bonds. Radical cations **5-10** and **5-9** through the SET step recovered Fe(III) to get into the next cycle.

### 2.2 Photoinduced copper-catalyzed fluoroalkylation

**SCHEME 6:**
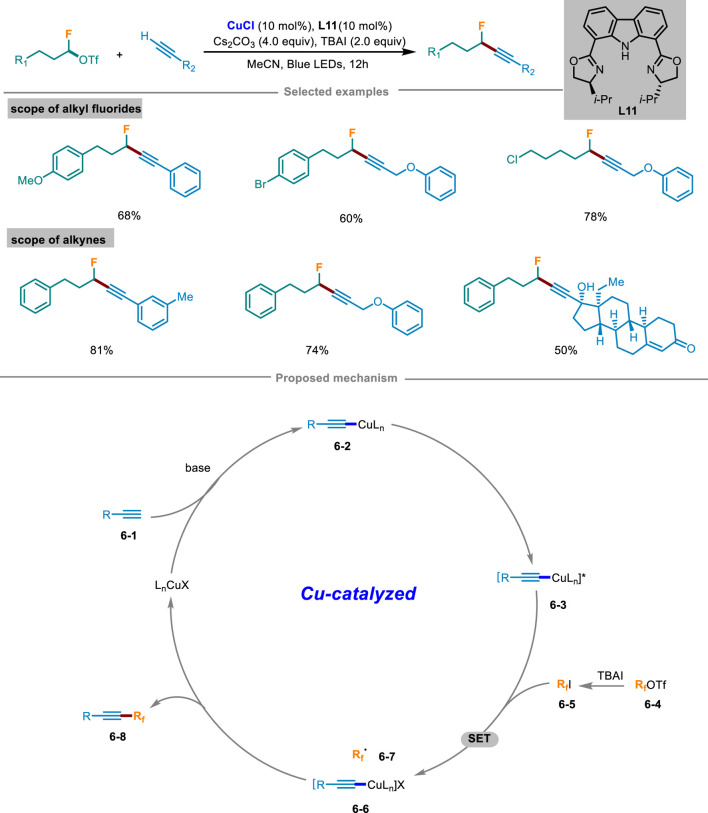
Photoinduced copper-catalyzed monofluoroalkylation of terminal alkynes.

In 2023, Wang and Li’s group disclosed a novel strategy of Cu(I) initiated cross-coupling reaction for the monofluoroalkylation of terminal alkynes under light irradiation (Scheme 6) (Hu et al., 2023). With readily available monofluoroalkyl trifluoromethanesulfonate as a fluorinating agent to avoid using highly toxic fluorinated reagents, this catalytic system has shown mild reaction conditions, a wide range of substrates, and strong compatibility of functional groups. This method could also apply to the late-stage modification of biologically active molecules. Control experiments exhibited that light irradiation, copper catalyst, and ligand **L11** were all crucial factors in the catalytic system. The reaction was completely inhibited by radical scavengers TEMPO or BHT and could detect TEMPO and BHT addition products by HRMS, which demonstrated that this catalytic cycle involved in a radical pathway. Based on these mechanistic experiments, a rationalized mechanism was illustrated in Scheme 6. At the beginning, L_n_CuX reacted with substrate alkyne **6-1** under the condition of base to afford Cu(I) complex **6-2**, which was then transformed into excited state **6-3** under light excitation. In the meantime, fluoroalkyl triflate **6-4** was converted to iodide **6-5** *in situ* in the existence of TBAI. Then the excited copper complex **6-3** and iodide **6-5** generated Cu(II) complex **6-6** and alkyl radical **6-7** through a SET process. Subsequently, the generated radical **6-7** was rapidly intercepted by **6-6** to yield the intended product **6-8**, and then regenerated Cu(I) species to continue the next copper catalytic cycle.

### 2.3 Cobalt-catalyzed fluoroalkylation by LMCT

**SCHEME 7:**
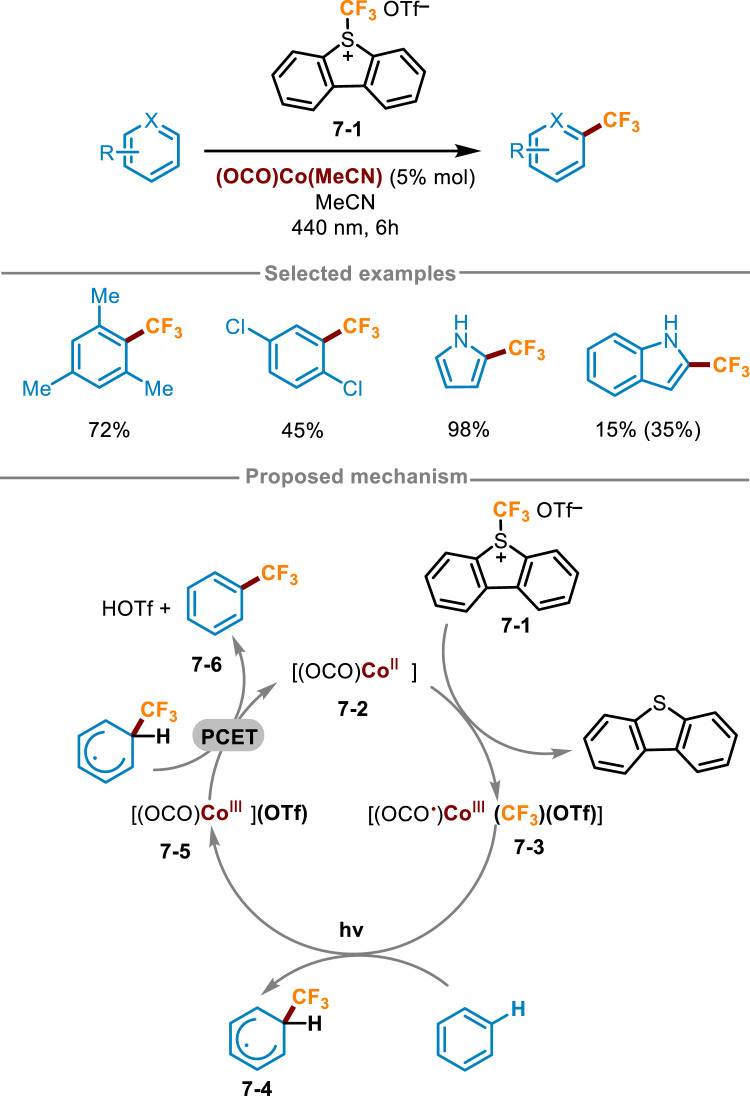
Cobalt photocatalyst for direct trifluoromethylation of (hetero)arene C (sp^2^)−H bonds.

Co complexes have also been studied as photocatalysts in organic synthesis. For example, in 2018, Zysman-Colman and Hanan synthesized [Co(dgpy)_2_]^3+^and [Co(dgpz)_2_]^3+^complexes, which served as very strong photo oxidants and can be applied in the trifluoromethylation of polycyclic aromatic hydrocarbons (Pal et al., 2018). In 2023, Kuehner and coworkers also achieved similar transformations using Co complexes (Scheme 7) (Kuehner et al., 2023). Unlike previous work, Kuehner’s work was achieved by the light induced activation of Co−CF_3_ intermediates, which were generated *in situ* and can simultaneously act as chromophores and organometallic reaction centers. Optimization of reaction conditions showed this reaction obtained a higher yield in the presence of (OCO)Co(MeCN) and Umemoto’s S-(trifluoromethyl)dibenzothiophenium triflate **7-1** with a 10-fold excess of reaction substrate in MeCN under irradiation with 440 nm blue LED for 6 h. The reaction yield decreased significantly when the electron cloud density of the ring was low. A workable mechanism was described in Scheme 7, [(OCO)Co^II^] **7-2** firstly reacted with Umemoto’s regent **7-1**, and then net CF_3_^+^ was added to Co(II) complex **7-2** to produce the photoactive (OCO ·)Co^III^(CF_3_) substance **7-3**. Next, visible light irradiation induced the homolysis of Co−CF_3_ bond of intermediates **7-3**, generating Co(III) intermediate **7-5** and trifluoromethyl radical. The radical was trapped by aromatic hydrocarbons to form the initial state cyclohexadiene radical **7-4**. Afterward, the expected product **7-6** was delivered through the net proton-coupled electron transfer (PCET) process between **7-4** and **7-5**, along with the regeneration of the Co(II) species to close the photoredox catalytic cycle.

### 2.4 Bismuth-catalyzed fluoroalkylation by LMCT

**SCHEME 8:**
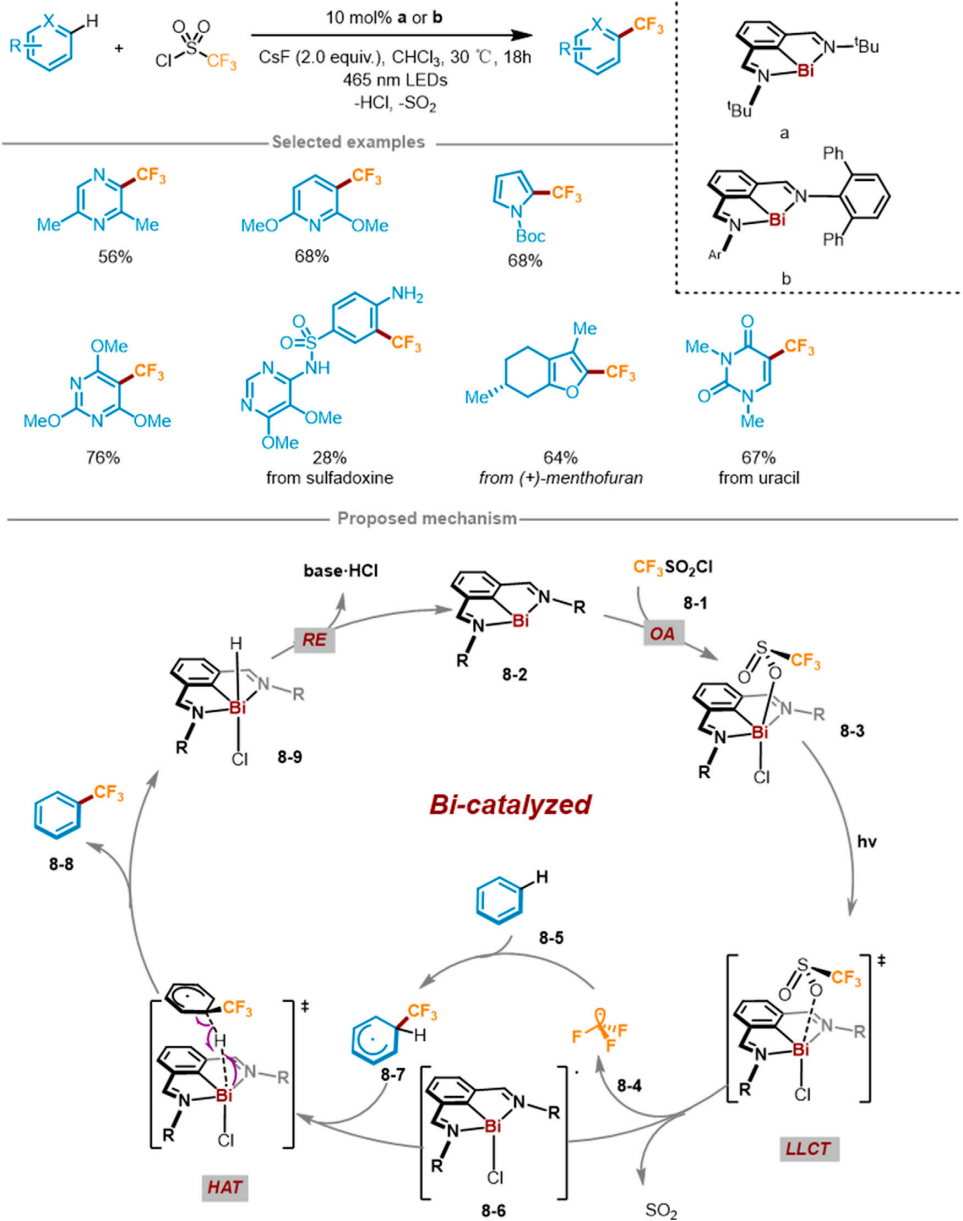
Bi-Catalyzed cycle of direct trifluoromethylation of (hetero)arenes under light.

In 2023, Josep Cornella and coworkers reported the trifluoromethylation of C(sp^2^)-H of (hetero)arenes harnessing Bi catalyst under visible light irradiation (Scheme 8) (Tsuruta et al., 2023). This reaction method utilized aromatics or heteroaromatics as substrates, CF_3_SO_2_Cl as CF_3_ reagent, 10 mol% Bi(I) as a catalyst, and 465 nm visible light in CHCl_3_ solvent at 30°C. The catalytic method can directly modify various heterocyclic aromatic hydrocarbons with different functional groups. Through ^19^F NMR and HRMS could observe the trifluoromethyl-TEMPO adduct, suggesting that generated trifluoromethyl radical upon light irradiation. The experiment with or without light proved that light irradiation was a necessary condition for Bi−O bond homolysis to generate CF_3_ radicals. According to mechanistic studies, a feasible catalytic cycle mechanism was formulated and presented in Scheme 8. Foremost, through radical oxidation addition (OA) of the fluorinating agent CF_3_SO_2_Cl **8-1** to Bi(I) catalyst **8-2**, the low-valent Bi(I) catalyst **8-2** was converted into Bi(III) intermediate **8-3**. Then the Bi−O bond of Bi(III) complex **8-3** was photoinduced homolysis through the ligand-to-ligand charge transfer (LLCT) process, producing CF_3_SO_2_ radical and Bi(II) radical **8-6**, followed by the release of CF_3_ radical **8-4** and SO_2_. Then CF_3_ radical **8-4** was added to (hetero)aromatic hydrocarbons to yield radical intermediate **8-7**. Through HAT process, the radical **8-6** extracted a hydrogen atom from **8-7** to rearomatize and deliver trifluoromethylation products **8-8** and Bi complex **8-9**. Finally, **8-9** released a molecule of hydrogen chloride through ligand coupling or alkali-promoted deprotonation to complete the catalytic cycle.

### 2.5 Silver-catalyzed fluoroalkylation by LMCT

**SCHEME 9:**
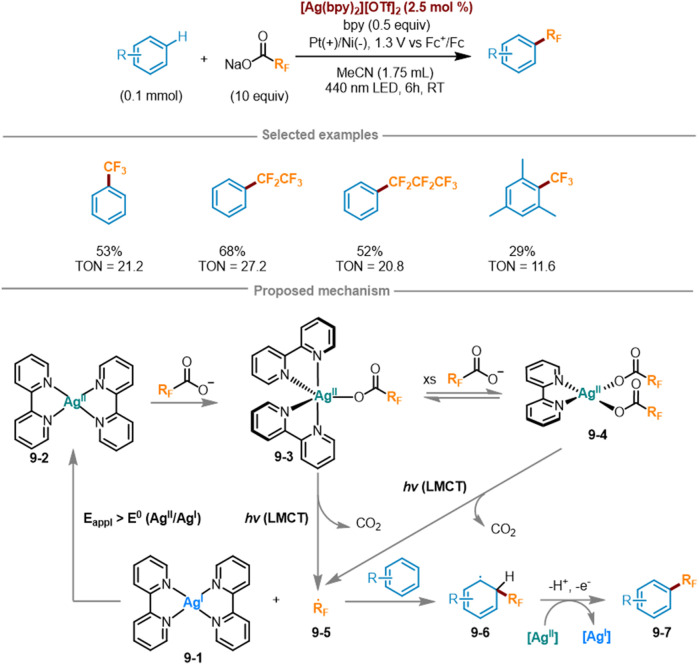
Ag (II)-mediated perfluoroalkylation of arenes via LMCT excitation under a catalytic cycle.

Due to the transfer of electron density towards the metal center, only electrophilic metals with high valence states can participate in LMCT photochemistry. Ag(I) is a strong oxidant [E° = 0.799 V vs. normal hydrogen electrode (NHE)], while Ag(II) has a d^9^ electron configuration and its 4d sublayer has empty orbitals, thus possessing stronger oxidation ability (E° = 1.980 V vs. NHE) (Grochala and Mazej, 2015). However, the electrophilic metals in the previous study did not include Ag(II). Based on the above situation, Daniel G. Nocera’s research group at *Harvard University* introduced a detailed introduction to a new electrooptic oxidation-reduction method in 2023 (Scheme 9) (Campbell et al., 2024), which utilized LMCT photochemistry coupled with Ag(II) and Ag(I) oxidation to transfer perfluoroalkyl radicals to aromatic hydrocarbons. This method used visible light to excite LMCT transitions to fill this empty orbital, Ag(II) metal centers can be utilized as photo oxidants to activate challenging substrates. This method can also be extended to other long-chain perfluoroalkyl carboxylates including R_F_CO_2_^–^. In a three-electrode split cell, under a bias voltage of 1.3 V versus Fc^+^/Fc, the trifluoromethylation of benzene was attained at 21.2 turnovers in the presence Na(TFA) as a limiting reagent (10 equiv.) and 2.5 mol% [Ag(bpy)_2_][OTf]_2_, 0.5 equivalent of bpy mixed with one equivalent of benzene for photoredox reaction (λ_exc_ = 440 nm). The bis (trifluoromethyl) benzene product can also be afforded with a yield of 11%. The possible rational mechanism was as follows: at an applied potential of [E_appl_ > E^o^ (Ag(II/I)], the low-valent Ag(I) complex **9-1** was oxidized to Ag(II) complex **9-2**. Subsequently, **9-2** combined with a fluoroalkyl carboxylate anion to yield Ag(II)-CO_2_R_F_ complex **9-3**, which may further react with another molecule of fluoroalkyl carboxylate anion to form Ag(bpy)(O_2_CCF_3_)_2_ **9-4.** Under excitation at a wavelength of 440 nm, both **9-3** and **9-4** was induced to the LMCT state, triggering photolysis and a rapid decarboxylation reaction to generate fluoroalkyl radicals **9-5** and CO_2_. Fluoroalkyl radicals **9-5** can easily engage in addition reactions with aromatic hydrocarbons to create C(sp^2^)-R_F_ bonds, generating aromatic radicals **9-6**, which were then oxidized by a second equivalent of Ag(II) to form fluorinated aromatic hydrocarbons **9-7**. Then the Ag(I) photoproduct underwent electrochemical reoxidation back to the Ag(II) photoreactant, achieving the photoelectrocatalytic cycle.

## 3 Conclusion and outlook

In the past decade, researchers have made significant progress in fluoroalkylation of various compounds via photocatalytic or electrochemical ways. In this review article, we take a comprehensive overview of recent research advancements concentrating on visible light-induced LMCT process to realize the fluoroalkylation of various substrates, such as alkenes, (hetero)arene, and terminal alkynes. The reactions were classified by different metal catalysts (Fe, Cu, Co, Bi, Ag). These schemes mostly utilize cheap and easily available fluoroalkyl carboxylic acids or these salts as reactants to introduce the required fluoroalkyl groups, including CF_3_, CF_2_H, CFHR, and C_m_F_n_ etc. They usually have mild conditions, high atomic economy, wide range, and potential ability of late-stage modification, which overcome the limitations of previous methods.

However, although significant progress being made in this field, considering the importance of the synthesis and research and development of fluorinated drugs, it still a crucial issue to continuously develop more economical and efficient strategies to introduce fluoroalkyl groups into compounds. At present, the substrates of LMCT-mediated fluoroalkylation strategies are limited to aryl and heteroaryl reactive systems, olefins. We believe that the LMCT-mediated strategy can expand the range of substrates, including more challenging inert compounds and valuable complex bioactive molecules. For instance, substrates can be expanded to carbonyl derivatives (such as aldehydes, ketones, alkyl aryl ketones, esters, and amides), polycyclic aromatic hydrocarbons (PAHs), benzyl compounds, and allyl compounds. These provide further opportunities for delivering the more valuable compounds with fluoroalkyl groups. In addition, LMCT-mediated asymmetric fluoroalkylation also needs to be further investigated. Photocatalytic fluoroalkylation by ligand-to-metal charge transfer is a relatively new research field, and we believe this review can provide valuable guidance for researchers in this field to stimulate the development of novel synthetic schemes for fluoroalkylation.
